# Real-World Data on Stage III Non-Small Cell Lung Cancer in Vietnam

**DOI:** 10.32604/or.2025.069281

**Published:** 2025-11-27

**Authors:** Khanh Toan Nguyen, Thi Huong Pham, Van Lam Ngo, Thi Thuy My Nguyen, Thi Dao Nguyen, Khanh Hung Truong, Van Nhat Nguyen, Van Thanh Le, Ba Duc Ho, Thi Phuong Thao Nguyen, Thi Ha Phuong Nguyen, Thi My Linh Dinh, Thi Hong Anh Vo, Thi Thuy Phan, Thi Hai Yen Le, Thi Nhung Ngo, Khanh Ha Nguyen

**Affiliations:** 1 Department of Medical Oncology 2, Nghe An Oncology Hospital, Vinh City, 43000, Vietnam; 2 Department of Oncology, Hanoi Medical University, Ha Noi City, 10000, Vietnam

**Keywords:** Non-small cell lung cancer, stage III, surgery, concurrent chemoradiotherapy, immunotherapy

## Abstract

**Objective:**

Patients with stage III non-small cell lung cancer (NSCLC) present with a heterogeneous disease profile and often require multifaceted treatment strategies. This research aimed to investigate the demographic features, therapeutic patterns, and survival outcomes of such patients in Vietnam.

**Methods:**

A retrospective descriptive study was conducted on 731 patients diagnosed with stage III NSCLC American Joint Committee on Cancer (AJCC) 8th edition, at Nghe An Oncology Hospital from January 2018 to August 2024. Descriptive statistics summarized baseline and treatment characteristics. We calculated progression-free survival (PFS) and overall survival (OS) through the Kaplan–Meier approach and compared survival curves with the log-rank test. Prognostic variables were assessed using Cox regression analysis.

**Results:**

Patients had a median age of 64 years, and the majority (84%) were male. Disease stages IIIA, IIIB, and IIIC accounted for 26.0%, 49.9%, and 24.1% of cases, respectively. Adenocarcinoma (60.7%) was the most common histological subtype. Initial treatments included surgery (8.5%), concurrent chemoradiotherapy (38.6%), sequential chemoradiotherapy (2.2%), radiotherapy alone (1.4%), systemic therapy (37.3%), and palliative care (12.0%). From 2018 to 2024, the use of systemic therapy declined (88.5% to 21.7%), while concurrent chemoradiotherapy rose significantly (1.1% to 51.5%). Median progression-free survival (mPFS) and median overall survival (mOS) were 8.9 months and 20.5 months, respectively. Patients with stage IIIA had significantly better outcomes (mPFS: 12.6 months; mOS: 32.4 months; *p* < 0.001). Surgical treatment yielded the longest survival (mPFS: 13.5 months; mOS: 42.8 months). Favorable prognostic factors included adenocarcinoma subtype, presence of driver mutations, stage IIIA, and good performance status.

**Conclusion:**

For stage III NSCLC, concurrent chemoradiotherapy is still considered the standard treatment, whereas surgery can provide the highest survival advantage in carefully selected cases. Histology, molecular profile, and disease stage are key prognostic indicators.

## 1 Introduction

Lung cancer remains one of the most significant causes of global cancer burden. In 2022, lung cancer ranked as the leading cancer worldwide in both new cases and deaths, with an estimated 2,480,301 incident cases (12.4% of all cancers) and 1,817,172 deaths (18.7% of all cancer-related deaths) [1]. In Asia, lung cancer was responsible for 1,142,397 deaths (62.9% of total cancer deaths) in 2022 [2]; a similarly high mortality rate was observed in Vietnam, with 22,597 deaths, accounting for 18.8% of cancer-related mortality [2]. Roughly 85% of lung cancer diagnoses are classified as non-small cell lung cancer (NSCLC) and are typically divided into three primary histological forms: adenocarcinoma, squamous cell carcinoma, and large cell carcinoma [3,4].

Recent trends show a higher proportion of early-stage diagnoses, with the 5-year survival rate improving notably to 26.4% [5]. Between one-fifth and one-third of lung cancer patients are diagnosed at stage III, the 5-year survival rate decreases from 36% in stage IIIA to 26% in stage IIIB, and further declines to 13% in stage IIIC [6]. Stage III NSCLC is considered a heterogeneous condition characterized by locally advanced tumors and/or mediastinal lymph node involvement without evidence of distant metastasis [7].

Management of stage III NSCLC requires multiple therapeutic strategies tailored to disease stage, leading to different management strategies and therapeutic outcomes depending on disease stage [8]. Multimodal treatment includes surgery, radiotherapy, and systemic therapies. For patients with resectable stage IIIA NSCLC, a combination of surgery and neoadjuvant therapy remains a potentially curative option [9]. In contrast, platinum-based concurrent chemoradiotherapy (cCRT) continues to be the cornerstone of treatment for individuals who are not candidates for surgery. Patients without disease progression after cCRT may benefit from consolidation with durvalumab, which has been shown to improve survival compared with cCRT alone [10–12]. More recently, osimertinib has provided additional benefit as consolidation therapy in patients with EGFR-mutated, unresectable stage III disease [13]. Other treatment approaches, including sequential chemoradiotherapy, chemotherapy or radiotherapy alone, and targeted agents, can be appropriate depending on patient characteristics and comorbidities [14]. Meanwhile, emerging strategies such as neoadjuvant chemo-immunotherapy have demonstrated promising early outcomes [15–17].

The relatively high prevalence of actionable gene mutations among Vietnamese lung cancer patients presents further opportunities for targeted therapy, contributing to improved quality of life and survival outcomes [18–20].

We performed a retrospective descriptive analysis to investigate the demographic characteristics, therapeutic patterns, and results among patients with stage III NSCLC in North-Central Vietnam’s clinical practice.

## 2 Subjects and Methods

### 2.1 Study Design and Patient Population

This retrospective descriptive study included 731 patients diagnosed with stage III NSCLC at Nghe An Oncology Hospital from January 2018 to August 2024. Diagnosis and treatment adhered to national guidelines and international standards [14,21,22]. Eligible patients had histologically confirmed NSCLC according to the WHO 2015 classification [23] and staged III based on the AJCC 8th edition guidelines [24]. Patients with unclear histology, multiple primary tumors, or incomplete records were excluded.

While positron emission tomography (PET/CT) and endobronchial ultrasound-guided transbronchial needle aspiration (EBUS-TBNA) are not available, staging procedures are carried out using the diagnostic modalities that are available at our institution, such as computed tomography (CT), magnetic resonance imaging (MRI), bone scan, and single photon emission computed tomography (SPECT/CT), which are routine [14].

### 2.2 Study Procedure

Patient data were collected retrospectively from medical records, with a cut-off date of 1 November 2024. Demographics, smoking status, comorbidities, TNM stage, histology, genetic mutation status, Eastern Cooperative Oncology Group performance status (ECOG PS), and initial treatment modality were among the extracted data. Prognostic factors for OS, overall survival (OS), and progression-free survival (PFS) were evaluated as outcomes.

Genetic testing was performed selectively, mainly for EGFR mutations via real-time Polymerase Chain Reaction (PCR), which is reimbursed by national insurance. Other mutations (ALK, ROS1, MET) were assessed using PCR or next-generation sequencing (NGS), depending on availability and patient affordability.

Treatment modalities were classified by primary therapeutic intent as surgery (with or without neoadjuvant/adjuvant therapy), concurrent chemoradiotherapy (cCRT), sequential chemoradiotherapy (sCRT), radiotherapy alone, or systemic therapy (chemotherapy, targeted therapy, or immunotherapy). In cases of combined treatment, classification was based on the primary therapeutic intent.

### 2.3 Study Variables

Progression-free survival (PFS) was measured from the start of treatment until disease progression, recurrence, or the end of the study period, whichever came first. Overall survival (OS) was determined from treatment initiation to death from any cause or the conclusion of the study.

Covariates included age (<70 vs. ≥70), sex, smoking status, ECOG PS (<2 vs. ≥2), histology (adenocarcinoma vs. others), stage (IIIA, IIIB, IIIC), mutation status (positive vs. negative), and comorbidities (yes vs. no). Comorbidities were grouped by organ system.

Comorbidities were categorized based on organ systems to facilitate analysis and interpretation. The major groups included cardiovascular diseases (e.g., hypertension, coronary artery disease), respiratory diseases (e.g., COPD), endocrine disorders (e.g., diabetes mellitus), hepatobiliary diseases, and musculoskeletal disorders. This grouping approach was adopted due to the wide range of comorbid conditions and their relatively low individual frequencies.

### 2.4 Statistical Analysis

Analyses were performed using SPSS v25.0, with *p* < 0.05 as the significance threshold. Survival outcomes were analyzed using Kaplan–Meier curves, with differences between groups assessed by the log-rank test. To identify independent prognostic factors, univariate and multivariate Cox proportional hazards regression analyses were applied. Variables with a *p*-value < 0.05 in the univariate analysis were subsequently included in the multivariate model. Additionally, a sensitivity analysis was performed, excluding patients whose mutation status was unknown.

### 2.5 Ethical Considerations

In line with the Declaration of Helsinki, the research protocol was examined and approved by the Ethics Committee of Nghe An Oncology Hospital (Decision No. 344/QD-BVUB, dated 28 February 2025).

As this was a retrospective study using anonymized medical records, informed consent was waived for all participants, including those who were deceased, in accordance with institutional ethical guidelines. All personal data was kept strictly confidential, used solely for research purposes, and no individual identifiers were disclosed.

## 3 Results

### 3.1 General Characteristics of the Study Population

The median age among the patients was 64 years (ranging from 26 to 88), with over 94% aged above 50. The predominant age bracket was 60–69 years (43.4%), with 26.3% aged more than 70 years. Males made up 84.0% of the cohort, and 74.1% had a smoking history, either former or current.

The majority of participants exhibited good performance status (PS 0–1) at 79.5%, and 62.9% had no comorbidities. Among those with comorbidities, cardiovascular diseases were the most frequent (17.9%), followed by respiratory diseases (7.9%).

Of the 731 patients included in the study, 190 (26.0%) were diagnosed with stage IIIA disease, 365 (49.9%) with stage IIIB, and 176 (24.1%) with stage IIIC. Among the entire cohort of 731 patients, only 279 (38.2%) underwent molecular testing. Of these, 111/279 patients (39.8%) tested positive for actionable driver mutations.

Regarding histology, adenocarcinoma was the predominant subtype (60.7%), followed by squamous cell carcinoma (28.9%). Only four cases (0.5%) were classified as large cell carcinoma, while the remaining cases (9.9%) were grouped under other histological subtypes.

During the study period from 2018 to 2024, there were no significant differences in demographic characteristics such as sex, age, performance status, or disease stage. However, there was a notable change in histological patterns: the proportion of adenocarcinoma in stage III NSCLC patients significantly increased from 44.8% to 69.7%. Conversely, the proportion of other histological types decreased from 25.3% to 6.1%, while the prevalence of squamous cell carcinoma showed a slight decline from 29.9% to 24.2%. The characteristics of the patients are summarized in Table 1.

**Table 1:** General characteristics of the study population

Characteristic		Value
Age median, years	Median (range)	64 (26–88)
Age group, *n* (%)	<50	42 (5.7)
	50–59	180 (24.6)
	60–69	317 (43.4)
	≥70	192 (26.3)
Sex, *n* (%)	Male	614 (84.0)
	Female	117 (16.0)
Smoking status, *n* (%)	Former/Current smoker	542 (74.1)
	Never smoked	189 (25.9)
ECOG Performance-status, *n* (%)	0–1	581(79.5)
	2	129 (17.6)
	3–4	21 (2.8)
Comorbidities (Frequency), *n* (%)*	Cardiovascular diseases	131 (17.9)
	Respiratory diseases	58 (7.9)
	Other	126 (17.2)
	No	460 (62.9)
Tumor histologic type, *n* (%)	Adenocarcinoma	444 (60.7)
	Squamous cell carcinoma	211 (28.9)
	Large cell carcinoma	4 (0.5)
	Other	72 (9.9)
Laterality, *n* (%)	Unknown	2 (0.3)
	Right	404 (55.3)
	Left	325 (44.5)
Stage, AJJC 8th edition, *n* (%)	III A	190 (26.0)
	III B	365 (49.9)
	III C	176 (24.1)
Genetic mutation, *n* (%)	Yes	111 (15.2)
	No	168 (23.0)
	Unknown	452 (61.8)

Note: *n*: number of patients; ECOG, Eastern Cooperative Oncology Group; AJCC, American Joint Committee on Cancer. *A patient may have more than one comorbidity; therefore, the percentages do not total 100%.

In addition, evaluation of tumor (T) and nodal (N) status revealed a heterogeneous distribution across the cohort. The majority of patients presented with T2–T4 tumors and N2–N3 nodal involvement, consistent with the locally advanced nature of stage III disease. A detailed breakdown of T and N classification is provided in Table S1.

### 3.2 Initial Treatment Modalities

Although stage III NSCLC is heterogeneous in terms of anatomical classification (T/N status) and therapeutic intent (curative vs. palliative), we chose to present our data primarily according to initial treatment modality. This approach reflects real-world clinical decision-making, where treatment choices are influenced by a combination of stage, patient performance status, comorbidities, molecular profile, and resource availability.

Furthermore, grouping by treatment allows a clearer comparison of clinical outcomes across modalities (e.g., surgery, chemoradiotherapy, systemic therapy), which aligns with our study’s aim of evaluating treatment patterns and real-world survival. To address potential selection bias, we provide detailed baseline characteristics by stage and other prognostic variables for each treatment group.

### 3.3 Surgical Treatment

Surgery was employed as the initial treatment modality in 8.5% of stage III NSCLC patients. Among these, 2 patients (3.2%) received neoadjuvant therapy prior to surgery, while 44 patients (71.0%) underwent adjuvant therapy postoperatively. The most common surgical procedure was lobectomy with regional lymph node dissection (*n* = 58, 93.5%), followed by segmentectomy and bilobectomy in a small number of cases.

### 3.4 Radiotherapy

Radiotherapy was utilized in 308 patients as part of the initial treatment approach. Among these, 96.8% received radical-intent radiotherapy, predominantly using intensity-modulated radiotherapy (IMRT). The standard curative dose was 60 Gy, delivered in 2 Gy fractions. A small proportion (3.2%) received radiotherapy alone, with a mean total dose of 54 Gy (2 Gy/fraction).

### 3.5 Systemic Therapy Chemotherapy

Chemot herapy was administered to 541 patients as part of initial treatment. Among them, 55.1% received chemotherapy in combination with definitive radiotherapy (concurrent chemoradiotherapy), while 44.9% received palliative chemotherapy. The most frequently used regimens in the concurrent setting were paclitaxel–carboplatin (42.6%), etoposide–cisplatin (32.2%), pemetrexed–platinum (20.1%), and vinorelbine–cisplatin (5.1%). For patients receiving chemotherapy alone, the most common regimens included paclitaxel–carboplatin (42.4%) and single-agent vinorelbine (17.7%).

### 3.6 Targeted Therapy

In patients with EGFR-mutated tumors, targeted therapy was administered either after surgery as adjuvant therapy, post-cCRT as consolidation, or as initial systemic treatment for those deemed inoperable. Specifically, 2 patients (5.9%) received adjuvant osimertinib following surgery, 3 patients (8.8%) received consolidation osimertinib post-cCRT, and 29 patients (85.3%) received EGFR-TKIs as primary treatment. The most commonly used agents were erlotinib (41.3%), gefitinib (31.0%), and afatinib (20.7%).

### 3.7 Immunotherapy

Immunotherapy was used either as consolidation or systemic therapy in selected patients. Eleven patients received durvalumab as consolidation following definitive cCRT, and one patient received pembrolizumab as first-line systemic therapy.

### 3.8 Initial Treatment Modalities

In our study, there were six primary treatment methods used for stage III lung cancer patients, including: surgery (8.5%), radiotherapy (1.4%), concurrent chemoradiotherapy (38.6%), sequential chemoradiotherapy (2.2%), systemic therapy (37.3%), and palliative therapy (12.0%). Curative surgery was primarily applied to stage IIIA patients with good performance status (26.3%). Concurrent chemoradiotherapy was used across all stages, most commonly in stage IIIB (43.8%). Systemic therapy was used most frequently in stage IIIC patients (52.3%). The other treatment methods, including radiotherapy, sequential chemoradiotherapy, and palliative therapy, showed similar rates across the different stages.

Concurrent chemoradiotherapy was the most common treatment method for both men (39.4%) and women (34.2%), followed by systemic therapy (38.4% for men and 31.6% for women). Among patients aged under 70 years, concurrent chemoradiotherapy was the predominant method (45.1%), while systemic therapy was most commonly used in patients aged 70 or older (51%). For patients with an ECOG performance status (PS) of <2, concurrent chemoradiotherapy (48.2%) and systemic therapy (32.5%) were the most commonly used methods. Conversely, systemic therapy (56.0%) and palliative therapy (36.7%) were the most prevalent methods for patients with an ECOG PS ≥ 2. Among subgroups based on comorbidities, smoking status, histology (adenocarcinoma or other types), and genetic mutation status, concurrent chemoradiotherapy and systemic therapy were the most commonly used treatments (Table 2).

**Table 2:** Distribution of prognostic factors among initial treatment groups

		Surgery n (%)	RT n (%)	cCRT n (%)	sCRT n (%)	Systemic Therapy n (%)	Palliative Therapy n (%)	Total *n* (%)	*p*
Stage	IIIA	50 (26.3)	2 (1.1)	68 (35.8)	5 (2.6)	47 (24.7)	18 (9.5)	190 (100.0)	**<**0.001
	IIIB	11 (3.0)	7 (1.9)	160 (43.8)	6 (1.6)	134 (36.7)	47 (12.9)	365 (100.0)	
	IIIC	1 (0.6)	1 (0.6)	54 (30.7)	5 (2.8)	92 (52.3)	23 (13.1)	176 (100.0)	
Sex	Male	44 (7.2)	7 (1.1)	242 (39.4)	13 (2.1)	236 (38.4)	72 (11.7)	614 (100.0)	0.041
	Female	18 (15.4)	3 (2.6)	40 (34.2)	3 (2.6)	37 (31.6)	16 (13.7)	117 (100.0)	
Age	<70	53 (9.8)	4 (0.7)	243 (45.1)	14 (2.6)	175 (32.5)	50 (9.3)	539 (100.0)	<0.001
	≥70	9 (4.7)	6 (3.1)	39 (20.3)	2 (1.0)	98 (51.0)	38 (19.8)	192 (100.0)	
Comorbidities	Yes	22 (8.1)	5 (1.8)	99 (36.5)	5 (1.8)	106 (39.1)	34 (12.5)	271 (100.0)	0.858
	No	40 (8.7)	5 (1.1)	183 (39.8)	1 (2.4)	167 (36.3)	54 (11.7)	460 (100.0)	
Smoking status	Yes	37 (6.8)	6 (1.1)	216 (39.9)	11 (2.0)	206 (38.0)	66 (12.2)	542 (100.0)	0.101
	No	25 (13.2)	4 (2.1)	66 (34.9)	5 (2.6)	67 (35.4)	22 (11.6)	189 (100.0)	
PS	<2	61 (10.5)	5 (0.9)	280 (48.2)	13 (2.2)	189 (32.5)	33 (5.7)	581 (100.0)	<0.001
	≥2	1 (0.7)	5 (3.5)	2 (1.3)	3 (2.0)	84 (56.0)	55 (36.7)	150 (100.0)	
Pathology	AC	50 (11.3)	2 (0.5)	177 (39.9)	14 (3.2)	152 (34.2)	49 (11.0)	444 (100.0)	<0.001
	Other	12 (4.2)	8 (2.8)	105 (36.6)	2 (0.7)	121 (42.2)	39 (13.6)	287 (100.0)	
Genetic mutation	Yes	19 (17.1)	1 (0.9)	44 (39.6)	2 (1.8)	41 (36.9)	4 (3.6)	111 (100.0)	<0.001
	No	17 (10.1)	4 (2.4)	63 (37.5)	6 (3.6)	66 (39.3)	12(7.1)	168 (100.0)	
	Unknown	26 (5.8)	5 (1.1)	175 (38.7)	8 (1.8)	166 (36.7)	72 (15.9)	452 (100.0)	

Note: RT, Radiotherapy; cCRT, Concurrent chemoradiotherapy; sCRT, Sequential chemoradiotherapy; AC, Adenocarcinoma; PS, performance status.

### 3.9 Trends in Disease Patterns over the Years

**Figure 1:**
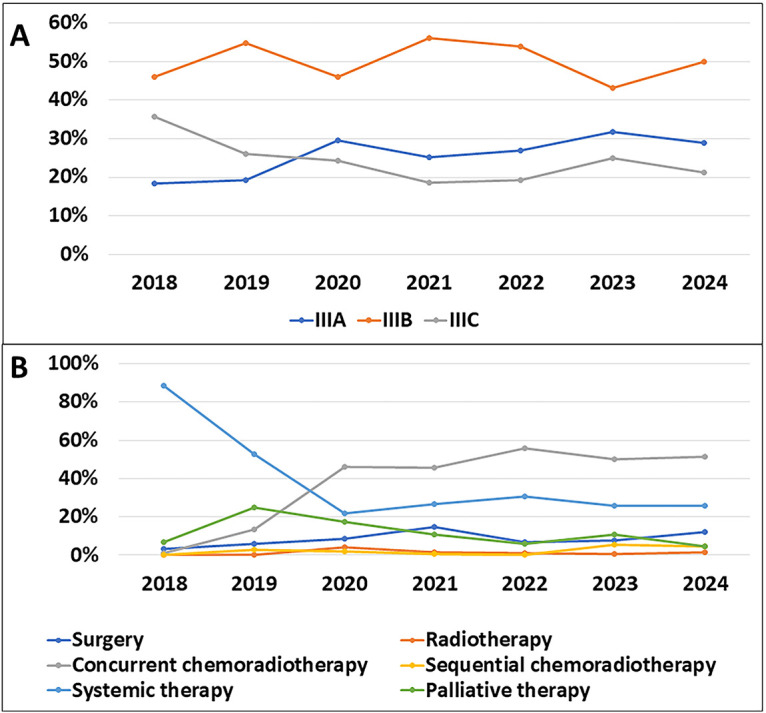
Temporal evolution of stage distribution and treatment selection during the study period. (**A**): New incidence rates by stage over the years. (**B**): Changes in treatment methods over the years

From 2018 to 2024, the incidence rate of stage IIIB disease was significantly higher than that of stage IIIA and IIIC. This rate exhibited considerable fluctuations, with a marked increase in 2019 and 2021, followed by a slight decrease and a subsequent increase again in 2024. The incidence of stage IIIA disease showed a rising trend from 2018 to 2020, followed by a slight decrease from 2020 to 2022, reaching its highest point of 31.8% in 2023. In contrast, the incidence of stage IIIC steadily decreased from 35.6% in 2018 to 21.2% in 2024 (Fig. 1A).

A graph illustrating the changes in treatment methods from 2018 to 2024 shows a significant shift in treatment preferences. In 2018, systemic therapy was the predominant treatment with a rate of 88.5%. However, this method saw a sharp decline over the years, dropping to just 25.8% in 2024. Concurrent chemoradiotherapy, on the other hand, exhibited a notable increase, rising from 1.1% in 2018 to 51.5% in 2024, making it the most commonly used treatment method. Surgery also increased from 3.4% in 2018 to 12.1% in 2024, reaching its peak in 2021 at 14.6%. Meanwhile, radiotherapy and sequential chemoradiotherapy remained relatively stable and consistently accounted for a small proportion of the treatments throughout this period (Fig. 1B).

### 3.10 Progression-Free Survival (PFS)

**Figure 2:**
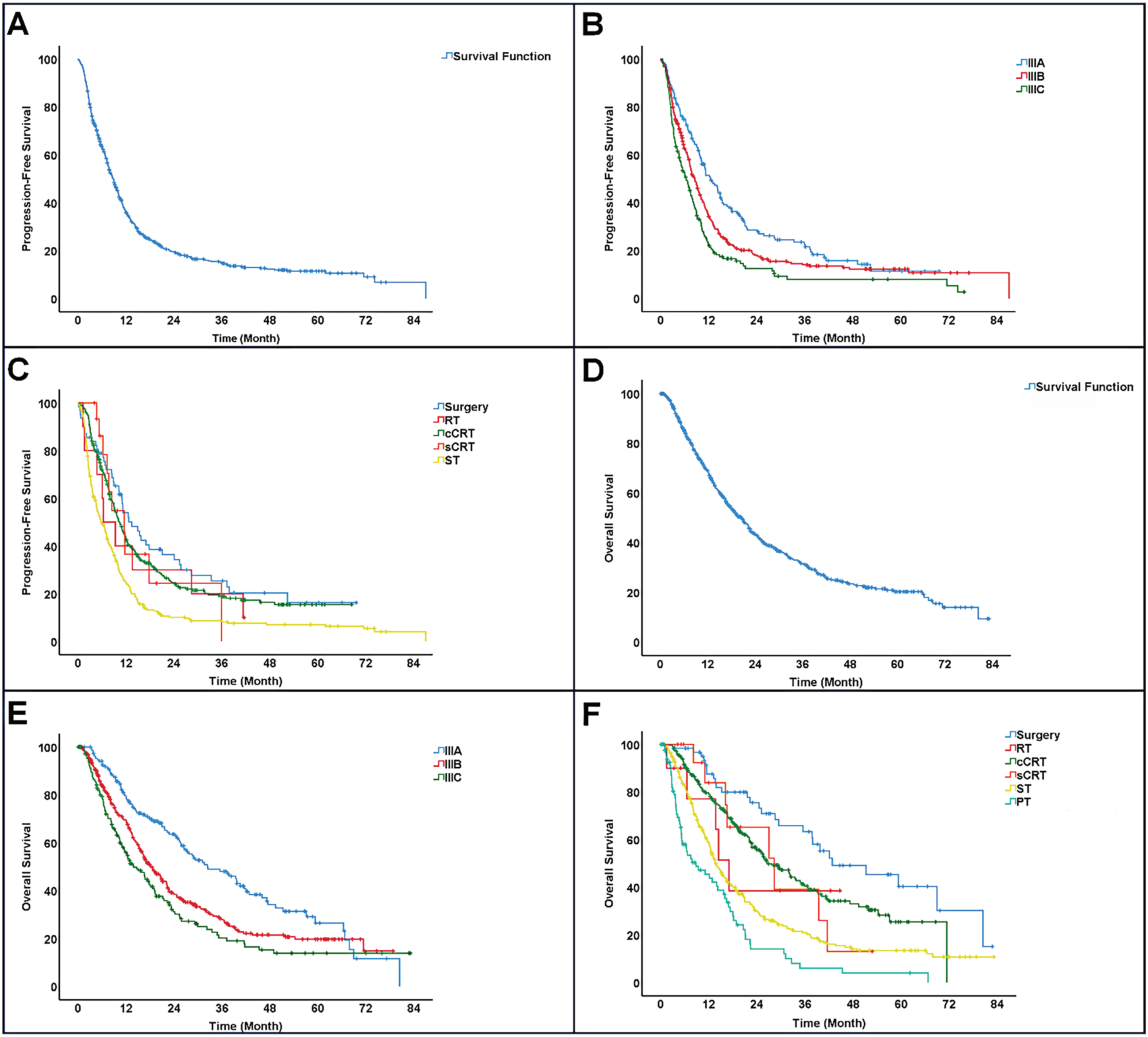
Survival outcomes of patients with stage III NSCLC. (**A**): Progression-free survival (PFS). (**B**): Progression-free survival (PFS) by disease stage. (**C**): Progression-free survival (PFS) by treatment. (**D**): Overall survival (OS). (**E**): Overall survival (OS) by disease stage. (**F**): Overall survival (OS) by treatment. Note: RT, Radiotherapy; cCRT, Concurrent chemoradiotherapy; sCRT, Sequential chemoradiotherapy; ST, Systemic therapy; PT, Palliative therapy

The median progression-free survival (PFS) was 8.9 months, with a 95% confidence interval (CI) ranging from 8.0 to 9.7 months. The PFS rates at 1, 2, 3, and 4 years were 35.9%, 19.3%, 14.7%, and 12.2%, respectively, while the 5-year PFS was not reached. Patients with stage IIIA disease had a median PFS of 12.6 months (95% CI: 10.2–15.0), which was significantly longer than those with stage IIIB (8.6 months; 95% CI: 8.4–9.7) and stage IIIC (6.3 months; 95% CI: 4.6–8.0) (*p* < 0.001) (Fig. 2A,B).

Treatment modalities showed varying median PFS outcomes: surgery yielded the longest at 13.5 months (95% CI: 8.7–18.2), followed by sequential chemoradiotherapy with 11.6 months (95% CI: 7.3–15.8), and concurrent chemoradiotherapy at 10.4 months (95% CI: 9.0–11.8). Patients treated solely with radiotherapy had a median PFS of 6.3 months (95% CI: 1.3–11.4), whereas those on systemic therapy alone had the shortest duration of 5.7 months (95% CI: 4.3–7.0). Surgical treatment appeared to offer the greatest benefit in prolonging progression-free survival (Fig. 2C).

### 3.11 Overall Survival (OS)

The median overall survival (OS) was 20.5 months (95% CI: 18.3–22.7). OS rates at 1, 2, 3, and 4 years were 68.8%, 43.3%, 31.4%, and 23.4%, respectively, with the 5-year OS not reached. OS varied significantly among different disease stages: patients with stage IIIA had a median OS of 32.4 months (95% CI: 23.6–41.2), compared to 18.1 months (95% CI: 15.6–20.6) for stage IIIB and 13.9 months (95% CI: 10.6–17.2) for stage IIIC (*p* < 0.001). The 5-year OS rate reached 26.5% only in stage IIIA patients (Fig. 2D,E).

The longest median overall survival (42.8 months; 95% CI: 22.7–62.9) was observed in patients who underwent surgery as their initial treatment, while those receiving only palliative care showed the shortest median OS of 8.8 months (95% CI: 2.9–14.8). Among other treatment modalities, sequential chemoradiotherapy was associated with a median OS of 28.3 months (95% CI: 13.6–43.0), closely followed by concurrent chemoradiotherapy at 26.5 months (95% CI: 21.7–31.4). Radiotherapy alone had a median OS of 17.1 months (95% CI: 12.7–21.5), and systemic therapy alone had the lowest median OS of 13.7 months (95% CI: 12.0–15.5). These results indicate that surgical intervention offers a marked survival benefit compared to other treatments (Fig. 2F).

### 3.12 The Relationship between the OS and Various Factors

Univariate analysis showed that a higher median overall survival (mOS) was significantly associated with female sex, age under 70 years, ECOG performance status <2, non-smoking status, adenocarcinoma histology, stage IIIA disease, presence of genetic mutations, and initial treatment with surgery, with all differences being statistically significant (*p* < 0.05). In the multivariate Cox regression model, four independent prognostic factors associated with a reduced risk of death were identified: good performance status (PS < 2), adenocarcinoma histology, stage IIIA disease, and presence of genetic mutations, all with statistical significance (*p* < 0.05). In contrast, sex, age, smoking status, and surgical treatment were not significantly associated with overall survival (Table 3). To further validate the prognostic role of driver mutations, a sensitivity analysis was performed after excluding patients with unknown mutation status. This analysis confirmed that mutation-positive patients had a significantly better OS than mutation-negative patients, supporting the robustness of mutation status as an independent prognostic factor (Table S2; Fig. S1).

**Table 3:** Univariate and multivariate analysis

Factor		Univariate		Multivariate	
		mOS (95% CI) (Months)	*p*	HR (95% CI)	*p*
Sex	Male	19.2 (16.9–21.6)	0.002	1	0.223
	Female	25.2 (11.2–39.1)		1.27 (0.87–1.85)	
Age	<70	22.2 (19.5–24.9)	0.010	1	0.937
	≥70	16.6 (13.2–20.0)		0.99 (0.78–1.26)	
Performance-status	PS < 2	22.8 (20.6–25.1)	<0.001	1	<0.001
	PS ≥ 2	12.2 (8.8–15.7)		0.54 (0.42–0.69)	
Smoking status	Yes	19.3 (16.6–22.0)	0.003		0.725
	No	22.4 (15.0–29.8)		1.06 (0.78–1.43)	
Pathology	AC	23.3 (19.2–27.4)	<0.001	1	0.039
	Other	15.5 (12.8–18.2)		0.81 (0.67–0.99)	
Stage	IIIA	32.4 (23.6–41.2)	<0.001	1	0.001
	IIIB/IIIC	16.9 (15.1–18.7)		0.67 (0.53–0.84)	
Genetic mutation	Yes	33.1 (22.5–43.7)	<0.001	1	0.027
	No/Unknown	18.7 (16.3–21.1)		0.70 (0.51–0.96)	
Treatment	Surgery	42.8 (22.7–62.9)	<0.001	1	0.056
	Other	18.6 (16.7–20.5)		0.66 (0.44–1.01)	

Note: mOS, median overall survival; HR, hazard ratio; CI, confidence interval; PS, performance status; AC, Adenocarcinoma. HR = 1.0 indicates the reference category in the multivariate Cox regression analysis.

## 4 Discussion

This real-world study analyzed treatment patterns and survival in 731 Vietnamese stage III NSCLC patients over six years. Our findings provide insights into the heterogeneity of this disease and reflect both local practices and global treatment trends.

The median age of 64 years (range: 26–88) observed in our study is lower than the global median age for lung cancer, which is approximately 71 years [25]. The majority of patients were older than 60 years (69.7%), predominantly male (84%), and had a history of smoking (74.1%). Adenocarcinoma was the most common histological subtype (60.7%), and the rate of genetic mutations in stage III patients was 39.8%. Compared to the KINDLE Asia study, the median age was similar at 63 years, while the proportions of male patients (74.8%), smoking history (72%), and adenocarcinoma subtype (55.7%) were slightly lower [26]. Recent studies from China further support the notion that NSCLC populations in Asia exhibit distinct demographic and molecular characteristics compared with Western counterparts [27].

The diversity in initial treatment approaches highlights the challenges posed by the heterogeneity of stage III NSCLC and the complexity of optimizing treatment decisions for individual patients. Similar patterns have been described in other Asian real-world cohorts. For example, the MOOREA study in China reported comparable diversity, with cCRT and systemic therapy as the most frequently adopted strategies [28]. In our study, the most common initial treatment was chemoradiotherapy (40.8%), primarily concurrent chemoradiotherapy (38.6%), applied across stages IIIA, IIIB, and IIIC with rates of 35.8%, 43.8%, and 30.7%, respectively. This was followed by systemic therapy at 37.3% (24.7% in stage IIIA, 36.7% in stage IIIB, and 52.3% in stage IIIC). These results are consistent with findings from the KINDLE Asia study [26] and are in line with general treatment guidelines [29]. Surgery was mainly employed for stage IIIA disease, with a rate of 26.3%, which is lower compared to the KINDLE Asia (37.5%) and KINDLE global (32%) studies. This difference may be attributed to the lower proportion of stage IIIA patients in our study compared to those studies [26,30].

There was also a notable shift in treatment trends for stage III NSCLC patients at our hospital over the study period. Specifically, the use of systemic therapy has decreased, while definitive, locoregional treatment modalities have become more prevalent. This trend mirrors those reported by other cancer centers and aligns with the global efforts to improve lung cancer control. According to an analysis conducted in the United Kingdom and Canada, the use of definitive treatments, particularly concurrent chemoradiotherapy, increased significantly between 2000 and 2007 among patients with stage III lung cancer [31]. Similarly, an analysis of the Surveillance, Epidemiology, and End Results (SEER) Program database showed a marked decrease in the proportion of patients diagnosed with distant metastatic disease between 2000 and 2022 [32]. International experience similarly shows that consolidation immunotherapy has transformed outcomes. Real-world studies have demonstrated that durvalumab following cCRT significantly improves survival in patients with unresectable stage III disease [11,33], thereby consolidating the PACIFIC trial findings and establishing immunotherapy consolidation as a global standard of care [34]. These trends likely reflect improvements in the TNM classification system, which has become more detailed and precise over time, as well as expanded access to advanced surgical and radiotherapy techniques, enabling more patients to benefit from curative-intent therapies. At our institution, diagnostic imaging capabilities have been gradually enhanced with the implementation of CT, SPECT/CT, MRI, and bone scintigraphy. However, the lack of PET-CT imaging and EBUS remains a limitation, potentially affecting the accuracy of staging and subsequently influencing the selection of optimal treatment strategies [35–37].

Previous reports, including the KINDLE Global and KINDLE Vietnam cohorts, demonstrated superior mPFS in stage IIIA compared with IIIB disease. Our findings align with this trend, with stage IIIA patients showing the most favorable survival outcomes (12.6 months), followed by IIIB (8.6 months) and IIIC (6.3 months) [30,38]. Regarding initial treatment modalities, patients undergoing surgery achieved a significantly longer mPFS of 13.5 months compared to non-surgical patients (*p* = 0.007). In our cohort, 26.3% of patients received curative surgery, primarily in stage IIIA. Concurrent chemoradiotherapy (cCRT) was the most commonly used initial therapy for stage III NSCLC at our institution, with administration rates of 35.8%, 43.8%, and 30.7% in stages IIIA, IIIB, and IIIC, respectively, and a steady increase from 2018 to 2024. Patients who underwent surgery had a median PFS of 13.5 months (95% CI: 8.7–18.2), notably longer than those receiving cCRT (10.4 months; 95% CI: 9.0–11.8) or sequential chemoradiotherapy (11.6 months; 95% CI: 7.3–15.8). These findings are consistent with results reported in the KINDLE Taiwan and KINDLE Vietnam studies [38,39].

The median overall survival (mOS) in our study was 20.5 months (95% CI: 18.3–22.7), which is lower than reported in KINDLE Asia (42.3 months; 95% CI: 38.1–46.8) and KINDLE Global (34.9 months; 95% CI: 32.0–38.0) [26,30]. This discrepancy likely reflects limited access to immunotherapy and advanced surgical or radiotherapy techniques in our setting. Recent perioperative immunotherapy trials, such as KEYNOTE-671 and CheckMate 816, have demonstrated substantial improvements in pathological response and survival [16,17]. Early-phase studies of atezolizumab also support the feasibility of IO-based neoadjuvant strategies [15,40]. Incorporating these advances into Vietnamese practice could help narrow the survival gap with international cohorts. In our cohort stage IIIA patients, the 5-year OS rate was 26.5%, whereas it remained unreached for stages IIIB and IIIC, possibly due to the relatively short median follow-up time. Significant differences in median OS were observed across stages: 32.4 months (95% CI: 23.6–41.2) for IIIA, 18.1 months (95% CI: 15.6–20.6) for IIIB, and 13.9 months (95% CI: 10.6–17.2) for IIIC (*p* < 0.001). Among a subgroup of 50 stage IIIA patients undergoing curative surgery, the longest median OS was observed at 42.8 months (95% CI: 22.7– 62.9), though this was lower than reported in a Korean cohort (52.5 months; 95% CI: 43.1–61.9) and KINDLE Asia (51.4 months; 95% CI: 43.8–64.1) [26,30]. The difference may be attributed to the smaller proportion of stage IIIA patients in our cohort (26%) compared to KINDLE Asia (54.7%).

For non-surgical patients receiving definitive chemoradiotherapy, median OS was 28.3 months (95% CI: 13.6–43.0) for sequential chemoradiotherapy, slightly higher than 26.5 months (95% CI: 21.7–31.4) for concurrent chemoradiotherapy, despite not being statistically significant (*p* = 0.747). This contrasts with the KINDLE Global study, which reported superior survival with concurrent chemoradiotherapy [30].

Among non-surgical candidates, survival outcomes were significantly better for patients treated with concurrent or sequential chemoradiotherapy compared to radiotherapy alone (median OS: 17.1 months; 95% CI: 12.7–21.5), systemic therapy (13.7 months; 95% CI: 12.0–15.5), or palliative care (8.8 months; 95% CI: 2.9–14.8) (*p* < 0.001).

Finally, when comparing treatment modalities, surgical patients had a significantly longer median OS of 42.8 months (95% CI: 22.7–62.9) vs. 18.6 months (95% CI: 16.7–20.5) for non-surgical patients (*p* < 0.001), consistent with a U.S. real-world study of 478 unresected stage III NSCLC patients reporting a median OS of 20 months [41]. Comparative real-world data reinforce this observation, showing that surgery confers a survival benefit in selected stage III patients [42]. Furthermore, emerging evidence suggests that neoadjuvant chemo-immunotherapy can downstage tumors and enable conversion surgery, offering promising outcomes for initially unresectable patients [43]. Retrospective analyses also suggest that chemo-immunotherapy followed by surgery may provide superior disease control compared with definitive CRT [44], supporting a paradigm shift toward multimodality regimens in resectable stage III NSCLC.

Statistical analyses confirmed independent prognostic factors linked with better survival outcomes, including ECOG performance status <2, adenocarcinoma histology, stage IIIA disease, and the presence of driver mutations. Patients with good PS <2 experienced a 46% reduction in mortality risk, aligning with findings from previous studies [6,31,39]. Adenocarcinoma histology was associated with a 19% survival advantage compared to other subtypes [6,31], potentially attributable to the higher prevalence of targetable mutations. Similarly, the presence of driver mutations conferred a 30% lower risk of death [26], underscoring the importance of molecular profiling even in stage III disease. Although female sex and younger age (<70 years) were associated with longer OS in univariate analysis, these factors were not retained in the multivariate models. Findings are consistent with KINDLE studies, which identified stage IIIA disease, good performance status, adenocarcinoma histology, and definitive treatment as favorable prognostic factors [26,30,39].

In multivariate analysis, ECOG PS < 2, stage IIIA disease, adenocarcinoma histology, and the presence of genetic mutations were identified as independent predictors of reduced mort. Surgery was associated with better OS but did not reach statistical significance (*p* = 0.056). No significant impact was observed for variables such as sex, age, and smoking history. This is consistent with the KINDLE studies, where stage IIIA, favorable performance status, adenocarcinoma subtype, and definitive therapies were identified as predictors of better outcomes [26,30,39].

### 4.1 Limitations

There are some important limitations to acknowledge. The retrospective, single-center approach may result in selection bias and reduce the extent to which these findings can be generalized to other regions in Vietnam or to healthcare settings in different countries. Second, although clinical and treatment data were collected comprehensively, genetic testing was performed in only a subset of patients, leading to potential misclassification when grouping mutation-negative and mutation-unknown cases. Third, treatment allocation was not randomized, and decisions were influenced by patient preferences, comorbidities, and local resource availability, which may have confounded survival outcomes. Fourth, information on certain prognostic variables, such as PD-L1 expression or detailed toxicity profiles, was unavailable, preventing further subgroup analyses. Finally, the relatively short median follow-up time for some patients may have led to an underestimation of long-term survival outcomes.

### 4.2 Implications

Despite these limitations, our findings provide valuable real-world evidence on treatment patterns and outcomes of stage III NSCLC in a Vietnamese setting. The results highlight the heterogeneity of treatment approaches, the underutilization of concurrent chemoradiotherapy, and the survival benefits of multimodal strategies, consistent with international studies. The relatively high prevalence of actionable mutations underscores the need to expand routine molecular testing to guide targeted therapy. The results can provide evidence to guide national health policies, optimize resource distribution, and support clinical decision-making, particularly in settings with constrained healthcare infrastructure. To validate these results and enhance treatment approaches for stage III NSCLC in Vietnam, future work should include multicenter prospective studies with extended follow-up and broad genetic testing to validate these findings.

## 5 Conclusion

Our research presents a detailed analysis of first-line treatment patterns for stage III NSCLC within Vietnam, representing real-world medical practices. Six distinct treatment modalities were identified, withconcurrent chemoradiotherapy remaining the most frequently applied approach. Patients who underwent surgery experienced better progression-free and overall survival compared to those receiving non-surgical therapies. Better outcomes were observed in patients with adenocarcinoma subtype, actionable genetic alterations, earlier stage IIIA disease, and preserved performance status (ECOG < 2).

## Supplementary Materials



## Data Availability

Upon reasonable request, the corresponding author will provide access to the data used in this study.

## Supplementary Materials

The supplementary material is available online at https://www.techscience.com/doi/10.32604/or.2025.069281/s1.
