# Supplementary material for: Real-World Data on Stage III Non-Small Cell Lung Cancer in Vietnam
Source: Oncol Res. 2025 Nov 27;33(12):4013–28. doi: 10.32604/or.2025.069281 (PMC12712671; doi:10.32604/or.2025.069281)
Supplement: Supplementary file 1 [file OncolRes-33-69281-s001.docx]

**Supplementary Materials:**

**Table S1:** Distribution of patients according to T and N stage classification.

| **Characteristic** | | **Value** |
| --- | --- | --- |
| Stage T, n (%) | T1 | 57 (7.8) |
|  | T2 | 145 (19.8) |
|  | T3 | 202 (27.6) |
|  | T4 | 319 (43.6) |
|  | Tx | 8 (1.2) |
| Stage N, n (%) | N0 | 67 (9.2) |
|  | N1 | 28 (3.8) |
|  | N2 | 349 (47.7) |
|  | N3 | 283 (38.7) |
|  | Nx | 4 (0.6) |

**Note:** T, primary tumor; N, regional lymph node; AJCC, American Joint Committee on Cancer (8th edition).

**Table S2:** Univariate analysis of genetic mutation status after excluding patients without molecular testing.

| **Factor** | | **Univariate** | |
| --- | --- | --- | --- |
|  |  | mOS (95% CI) (months) | *p* |
| Genetic mutation | Yes (n = 111) | 33.1 (22.5 – 43.7) | 0.005 |
|  | No (n= 168) | 21.6 (15.0 – 28.2) |  |

**
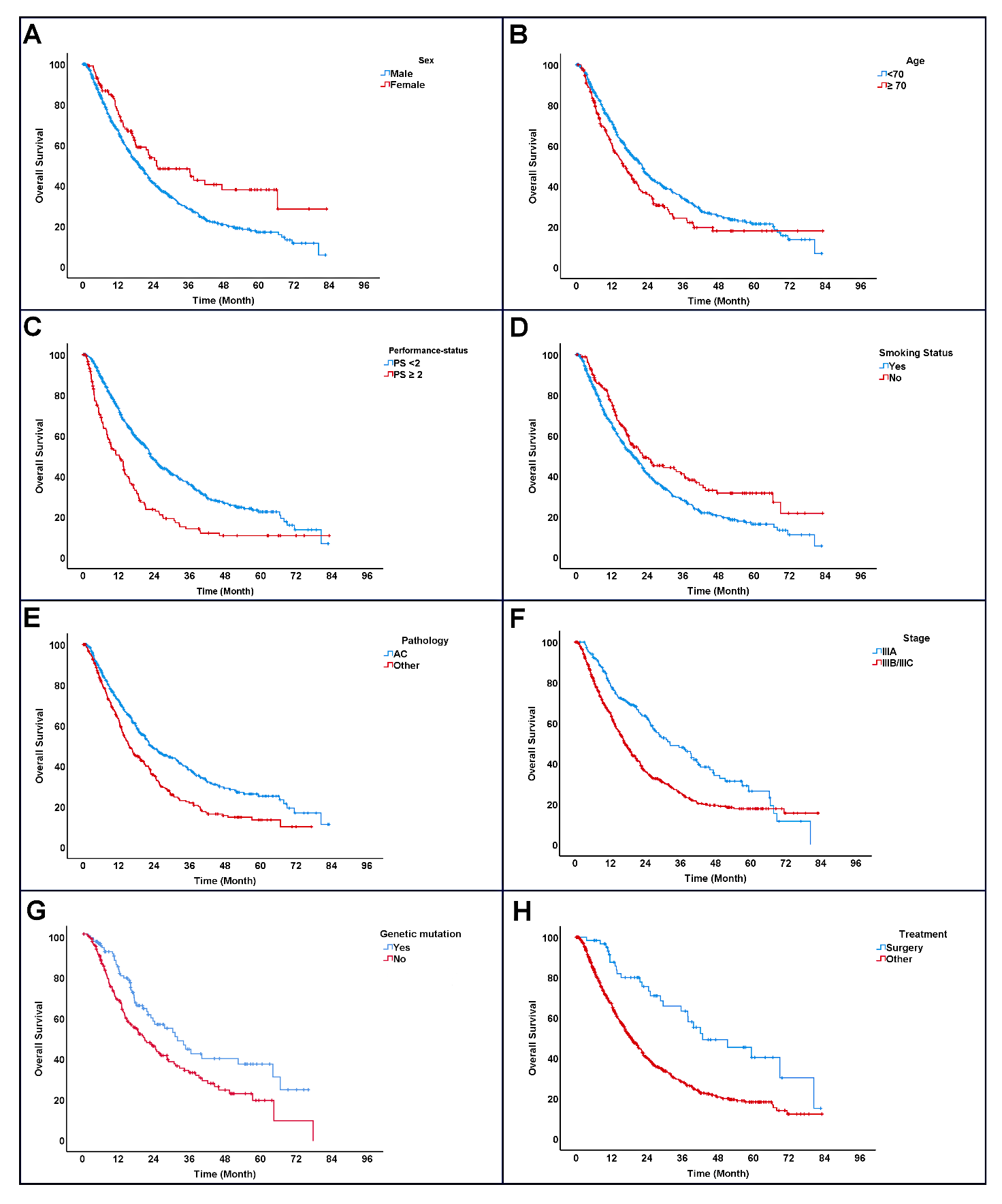
Figure S1:** Overall survival (OS) stratified by baseline clinical and molecular characteristics. (A). Overall survival (OS) by Sex. (B) Overall survival (OS) by Age. (C) Overall survival (OS) by Performance-status. (D) Overall survival (OS) by Smoking Status. (E) Overall survival (OS) by Pathology. (F) Overall survival (OS) by Stage. (G) Overall survival (OS) by Genetic mutation. (H) Overall survival (OS) by Treatment.
